# The Metabolic Regulation of Antioxidant Defense: Exogenous Ascorbate Disrupts Redox Homeostasis Under Energy Limitation in *Bangia fuscopurpurea*

**DOI:** 10.3390/plants15081165

**Published:** 2026-04-09

**Authors:** Hongting Xue, Xiaoxi Lin, Zhourui Liang, Yanmin Yuan, Chenchen Sun, Xiaoping Lu, Wenjun Wang

**Affiliations:** 1 National Demonstration Center for Experimental Fisheries Science Education, Shanghai Ocean University, Shanghai 201306, China; 2 State Key Laboratory of Mariculture Biobreeding and Sustainable Goods, Yellow Sea Fisheries Research Institute, Chinese Academy of Fishery Sciences, Qingdao 266071, Chinaliangzr@ysfri.ac.cn (Z.L.); yuanyanmin001@gmail.com (Y.Y.);; 3 Laboratory for Marine Fisheries Science and Food Production Processes, Laoshan Laboratory, Qingdao 266237, China

**Keywords:** *Bangia fuscopurpurea*, energy limitation, sub-saturating light, ascorbic acid, AsA-GSH cycle

## Abstract

*Bangia fuscopurpurea* is a marine alga with significant commercial value. Although a high-light adapted species, the productivity of its commercial cultivation is frequently limited by environmental light attenuation, resulting in the algae operating under energy-limiting, sub-saturating conditions. This study investigated its physiological responses and antioxidant defense mechanisms across a sub-saturating light gradient (20, 40, and 80 µmol photons m^−2^ s^−1^). We employed exogenous ascorbic acid (AsA) supplementation to evaluate the dynamic response of the ascorbate-glutathione (AsA-GSH) cycle. Without AsA supplementation, the 40 µmol photons m^−2^ s^−1^ condition supported redox homeostasis and the highest soluble protein accumulation. In contrast, the lowest irradiance (20 µmol photons m^−2^ s^−1^) restricted physiological performance. At 80 µmol photons m^−2^ s^−1^, which remained below the light saturation point, the algae experienced oxidative stress, indicated by elevated lipid peroxidation and hydrogen peroxide levels. The efficacy of exogenous AsA depended on these energy states. Under the highest tested irradiance (80 µmol photons m^−2^ s^−1^), AsA reduced malondialdehyde (MDA) and maintained electron transport capacity, but these effects were accompanied by a significant degradation of photosynthetic pigments. These findings imply an altered partitioning of cellular reducing power, where the demand for AsA regeneration might limit the resources available for biosynthetic pathways. The study highlights that antioxidant efficacy is constrained by the cellular energy availability, which limits simultaneous stress mitigation and growth in light-limited aquaculture environments.

## 1. Introduction

*Bangia* is a primitive red algal genus comprising both freshwater and marine species. It has evolved over at least 1.2 billion years and serves as a model for studying the evolution of multicellularity and complex life cycles in red algae [1]. *Bangia fuscopurpurea*, a marine species, exhibits a broad global distribution ranging from 70° N to 43° S, spanning subboreal to subtropical regions, growing on stony surfaces in high intertidal zones exposed to strong wave action [2,3]. In China, marine populations extend from Hong Kong to Tianjin, whereas freshwater populations are widely distributed across inland provinces such as Shanxi, Yunnan, and Tibet [4]. Despite its global ecological relevance, its large-scale commercial cultivation has currently only been reported in China [5]. It has been farmed there since the 1980s, providing a key system for investigating the species’ physiological constraints under intensive farming. Putian, located in Fujian province, serves as the primary production area. Currently, more than 67 hectares (1000 mu) are under cultivation, mainly on Nanri Island and Meizhou Island in Putian. Aquaculture of *B. fuscopurpurea* combines economic value with ecological sustainability and has become one of the important landmark marine economic industries in the local area.

Light intensity is a crucial environmental factor that significantly affects the photosynthetic efficiency and overall physiological functions of algae, influencing their distribution and stress responses [6]. Insufficient irradiance restricts photosynthetic activity and impairs growth. Conversely, excessive light overstimulates the photosynthetic apparatus, leading to an overproduction of reactive oxygen species (ROS) and subsequent oxidative stress [7]. Under light stress, a balance is maintained between photosynthetic efficiency and the activation of antioxidant defense systems in marine algae to maintain cellular homeostasis and mitigate oxidative damage [8]. These defense systems, both enzymatic and non-enzymatic, act synergistically to neutralize ROS. Enzymatic systems, such as superoxide dismutase (SOD), catalase (CAT), ascorbate peroxidase (APX), and glutathione peroxidase (GPX), convert ROS to less harmful molecules. Meanwhile, non-enzymatic antioxidants, including reduced glutathione (GSH), ascorbic acid (AsA), carotenoids (Car), and vitamin E, scavenge ROS through redox reactions [9].

Ascorbic acid (AsA) functions as a primary water-soluble antioxidant and an integral component of the photosynthetic electron transport network [10,11,12]. Mechanistically, AsA serves as the specific electron donor for ascorbate peroxidase (APX) within the water–water cycle (Mehler-peroxidase reaction) [13]. In this pathway, electrons derived from Photosystem I reduce dioxygen to water via AsA, thereby dissipating excess excitation energy. Additionally, AsA acts as an essential cofactor for violaxanthin de-epoxidase (VDE) [14], facilitating the conversion of violaxanthin to zeaxanthin and the induction of non-photochemical quenching (NPQ) for thermal energy dissipation [15]. The continuous regeneration of oxidized AsA requires reducing equivalents, primarily NADPH [16,17,18]. Consequently, AsA metabolism is stoichiometrically coupled to the cellular energy balance, as antioxidant regeneration competes with carbon assimilation for available photoreductants. However, the biological activity of AsA is complex; under certain conditions, such as the presence of transition metal ions or redox imbalance, AsA can also exhibit pro-oxidant properties. This can potentially catalyze the formation of highly reactive hydroxyl radicals [19,20].

AsA has been shown to significantly enhance abiotic stress tolerance in plants and algae by boosting antioxidant systems, modulating redox equilibrium, improving physiological metabolism, and interacting with various stress-response pathways [21,22]. For example, in maize, exogenous AsA enhances APX activity and restores the GSH/oxidized glutathione (GSSG) ratio. This regulation effectively reduces membrane lipid peroxidation and mitigates damage caused by ROS under salt stress conditions [23]. In *Fraxinus mandshurica*, exogenous AsA significantly improves the efficiency of somatic embryogenesis induction. By precisely regulating the AsA/dehydroascorbic acid (DHA) redox pair and APX activity, AsA inhibits polyphenol oxidase-mediated browning caused by oxidative stress during in vitro culture [24]. Furthermore, in *Pyropia yezoensis*, exogenous application of 1-aminocyclopropane-1-carboxylic acid (ACC) promotes AsA synthesis, which synchronizes algal development and stress responses, an effect comparable to that of exogenously supplied AsA [25]. Elucidating how these metabolic demands are coordinated in *B. fuscopurpurea* is critical for understanding its stress response mechanisms. This knowledge provides a basis for identifying specific, targetable pathways to enhance the resilience of this commercially important alga in fluctuating aquaculture environments.

*Bangia fuscopurpurea* is a typical high-intertidal red alga with a robust constitutive antioxidant system that enables it to survive variable environmental fluctuations [4,26,27,28,29,30,31]. In these regions, tidal-induced sediment resuspension and terrestrial discharge significantly increase water turbidity, leading to substantial attenuation of underwater irradiance [32,33]. Furthermore, the high stocking density within cultivation facilities results in a reduction of available light per unit of algal biomass [7,34,35]. These environmental factors collectively cause the majority of the harvestable biomass to operate under sub-saturating light conditions for extended periods. Therefore, our experimental gradient (20–80 µmol photons m^−2^ s^−1^) was designed to represent the physiological state of the bulk biomass under such energy-limited conditions. Understanding the metabolic shifts in this regime is essential for optimizing cultivation strategies in intensive mariculture.

While previous studies have primarily investigated responses to salinity stress [28,29,36], the metabolic demand of sustaining antioxidant defense systems under chronic energy limitation remains poorly characterized. This study shifts the analytical focus from general antioxidant capacity to energetic expenditure. Specifically, the mechanisms underlying the energy distribution between the energetic demand of the ascorbate-glutathione (AsA-GSH) cycle and other metabolic functions under restricted NADPH supply are unknown. This study investigated the physiological state and AsA-GSH cycle dynamics across a sub-saturating light gradient (20, 40, and 80 µmol photons m^−2^ s^−1^). Exogenous AsA supplementation was applied to impose a specific redox load, thereby evaluating the cellular ability to support antioxidant regeneration.

We hypothesized that distinct physiological states exist within the sub-saturating regime, with 40 µmol photons m^−2^ s^−1^ representing a state of metabolic equilibrium. Irradiances below and above this level were expected to induce source-limited energy deficits and sink-limited metabolic imbalances, respectively. Furthermore, exogenous AsA was predicted to function as a metabolic sink for reducing power, altering redox homeostasis under energy limitation while inducing competitive metabolic inhibition at higher sub-saturating irradiances.

## 2. Results

### 2.1. Chlorophyll Fluorescence Responses to Light Intensity and Exogenous AsA Treatment

**Figure 1:**
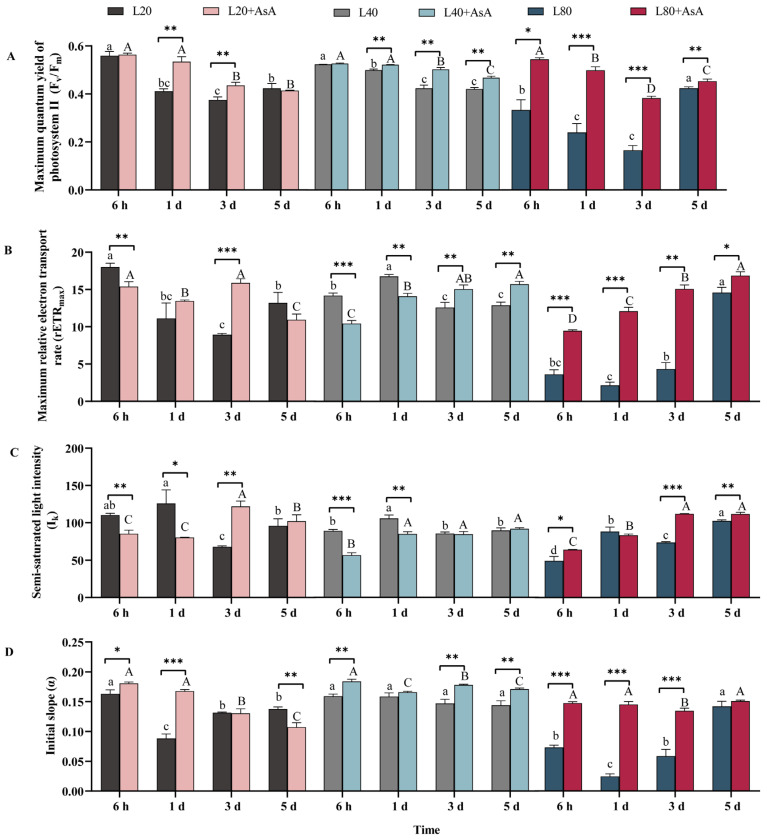
Effects of light intensity and AsA on chlorophyll fluorescence parameters in *B. fuscopurpurea* assessed by the IMAGING-PAM technique. The figure displays changes in (**A**) maximum quantum yield of photosystem II (F_v_/F_m_), (**B**) maximum relative electron transport rate (rETR_max_), (**C**) semi-saturated light intensity (I_k_), and (**D**) initial slope (α) over five days. Data are presented as means ± SD (*n* = 3). Different lowercase (a–d) and uppercase (A–D) letters indicate significant temporal differences among the four time points (6 h, 1 d, 3 d, and 5 d) for the −AsA and +AsA groups, respectively, within the same light intensity (one-way ANOVA, Tukey’s HSD, *p* < 0.05). Asterisks indicate significant differences between the −AsA and +AsA treatments under identical light and time conditions (independent-samples *t*-test; * *p* < 0.05, ** *p* < 0.01, and *** *p* < 0.001). The main and interactive effects of light intensity, AsA, and time are detailed in Table S1.

Light intensity and exogenous AsA drove distinct temporal patterns in the chlorophyll fluorescence parameters of *B. fuscopurpurea* (Figure 1). As detailed in Table S1, light intensity, AsA treatment, and time had significant main and interactive effects on these parameters (*p* < 0.001). In the absence of AsA, L40 generally sustained higher photosynthetic efficiency, particularly regarding maximum quantum yield of photosystem II (F_v_/F_m_) (Figure 1A) and the initial slope (α) (Figure 1D) over the 5 days compared to L20 and L80. Conversely, under L80, three photosynthetic parameters (F_v_/F_m_, maximum relative electron transport rate (rETR_max_), and semi-saturated light intensity (I_k_)) exhibited similar dynamics (Figure 1A–C), showing significant recovery by day 5 following an initial suppression at 6 h and 1 d.

Exogenous AsA application modulated these responses. Notably, it consistently enhanced rETR_max_ under L80 across all time points, reaching the highest observed value among all treatments by day 5 (Figure 1B). Under L20, AsA exerted a biphasic effect on rETR_max_. Regarding F_v_/F_m_, supplementation induced an increase under L80 during the initial three days. By day 3, this positive effect extended across all light treatments, but it eventually diminished by day 5 (Figure 1A). Furthermore, AsA increased I_k_ under L80 while initially suppressing it at L20 and L40 (Figure 1C). An initial enhancement of α occurred at 6 h across all irradiances following AsA addition, persisting most prominently under L40 (Figure 1D).

### 2.2. Light-Dependent Regulation of Chlorophyll-a and Carotenoid Accumulation and the Role of Exogenous AsA

**Figure 2:**
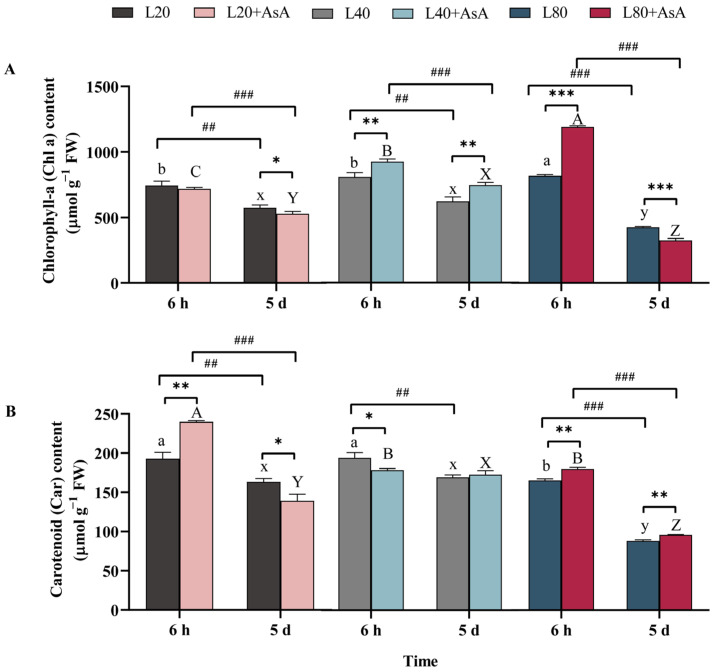
Pigment content of *B. fuscopurpurea* under different light intensities and exogenous AsA treatments. The figure shows (**A**) chlorophyll-a (Chl a) content and (**B**) carotenoid (Car) content. Data are presented as means ± SD (*n* = 3). Different letters indicate significant differences among light intensities within the same treatment and time point (one-way ANOVA, Tukey’s HSD, *p* < 0.05): lowercase (a, b for 6 h; x, y for 5 d) for the −AsA group, and uppercase (A–C for 6 h; X–Z for 5 d) for the +AsA group. Asterisks (*) denote significant differences between the −AsA and +AsA treatments, while hash marks (#) indicate significant temporal differences between 6 h and 5 d under identical conditions (independent-samples *t*-test; * *p* < 0.05; **, ## *p* < 0.01; ***, and ### *p* < 0.001). The detailed statistical results of the three-way ANOVA regarding the main and interactive effects are provided in Table S2.

As detailed in Table S2, the accumulation of photosynthetic pigments in *B. fuscopurpurea* was significantly influenced by the interactive effects of light intensity, exogenous AsA, and exposure time. Without AsA supplementation, the accumulation was dependent on light intensity, with L40 maintaining the highest pigment concentrations over the experimental period. For chlorophyll-a (Chl a) (Figure 2A), a significant light-dependent pattern was observed. At 6 h, contents were significantly higher under the L80 conditions compared to the L20 and L40 conditions (*p* < 0.05). By 5 d, L20 and L40 still maintained the highest level, whereas Chl a in the L80 group declined and became the lowest among all light treatments (*p* < 0.05). Carotenoid (Car) content exhibited a distinct pattern, maintaining significantly higher levels under L20 and L40 compared to L80 at both 6 h and day 5 (*p* < 0.05; Figure 2B).

Exogenous AsA application elicited dynamic pigment responses. Initial supplementation (at 6 h) significantly increased Chl a content under L40 and L80 (*p* < 0.05 and *p* < 0.01, respectively) without affecting the L20 group. Prolonged exposure to AsA (day 5) enhanced Chl a strictly under L40, while inducing a significant reduction under both L20 and L80 (*p* < 0.05). Regarding carotenoids, AsA initially stimulated accumulation under L20 and L80 but caused a reduction under L40 at 6 h. By day 5, AsA specifically suppressed Car content under L20. Overall, exogenous AsA provided an early phase enhancement of pigment levels at L20 and L80, whereas prolonged exposure resulted in diminished or reversed effects.

### 2.3. Oxidative Damage and Protein Response Under AsA Supplementation

**Figure 3:**
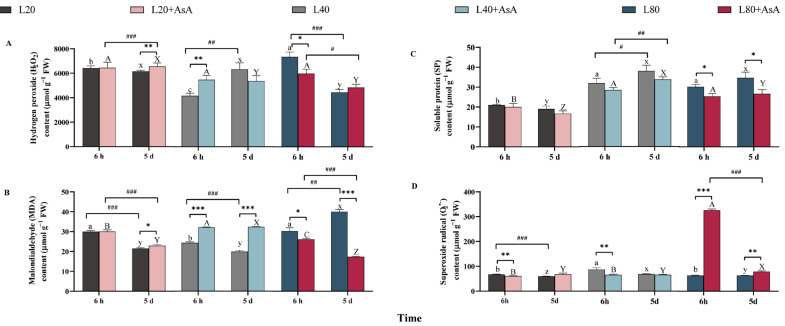
Changes in oxidative stress markers and soluble protein content in *B. fuscopurpurea*. The figure shows (**A**) hydrogen peroxide (H_2_O_2_) content, (**B**) malondialdehyde (MDA) content, (**C**) soluble protein (SP) content, and (**D**) superoxide anion (O_2_^•−^) content. Data are presented as means ± SD (*n* = 3). Different letters indicate significant differences among light intensities within the same treatment and time point (one-way ANOVA, Tukey’s HSD, *p* < 0.05): lowercase (a–c for 6 h; x–z for 5 d) for the −AsA group, and uppercase (A–C for 6 h; X–Z for 5 d) for the +AsA group. Asterisks (*) denote significant differences between the −AsA and +AsA treatments, while hash marks (#) indicate significant temporal differences between 6 h and 5 d under identical conditions (independent-samples *t*-test; *, # *p* < 0.05; **, ## *p* < 0.01; ***, and ### *p* < 0.001). The detailed statistical results of the three-way ANOVA regarding the main and interactive effects are provided in Table S2.

In *B. fuscopurpurea*, the responses of hydrogen peroxide (H_2_O_2_), malondialdehyde (MDA), superoxide anion (O_2_^•−^), and soluble protein (SP) revealed dynamic temporal patterns driven by the complex interactions among light intensity and exogenous AsA supplementation (Table S2). Without AsA supplementation, the highest H_2_O_2_ accumulation occurred under L80 at 6 h, followed by a significant decrease to the lowest observed level by day 5 (*p* < 0.001; Figure 3A). Initial lipid peroxidation (MDA) at 6 h was significantly higher under L20 and L80 compared to L40. Over the five-day period, MDA levels exhibited a significant decline under L20 and L40 (*p* < 0.001; Figure 3B), whereas prolonged L80 exposure induced substantial MDA accumulation (*p* < 0.01). The O_2_^•−^ response was distinct, peaking under L40 at 6 h; O_2_^•−^ content subsequently decreased significantly in the L20 and L40 groups by 5 d but remained unchanged in the L80 group (Figure 3D).

Exogenous AsA modulated these oxidative markers depending on the irradiance. Under L80, AsA reduced H_2_O_2_ at 6 h and significantly lowered MDA at both time points (*p* < 0.05). However, these reductions coincided with a transient 5.18-fold increase in O_2_^•−^ at 6 h, which subsequently dropped to approximately 24% of its peak level by day 5 (*p* < 0.001). In contrast, AsA addition under L40 resulted in elevated H_2_O_2_ content at 6 h and significantly higher MDA levels by day 5. Concurrently, SP accumulation exhibited a non-significant downward trend at both time points (Figure 3C).

### 2.4. GSH-GSSG Pool Dynamics and Redox State in Response to Light and AsA Treatments

**Figure 4:**
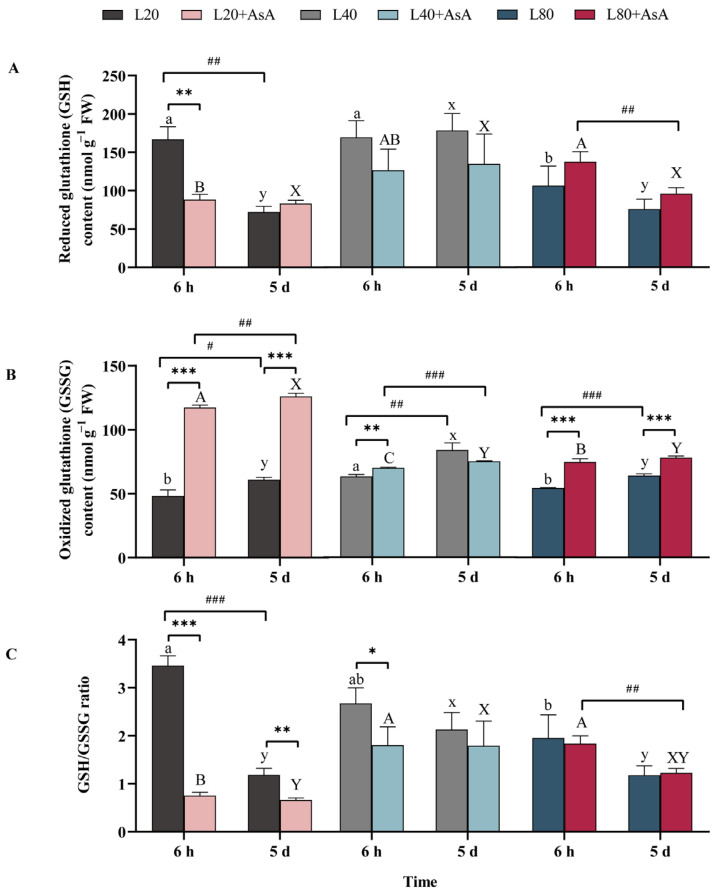
Changes in the glutathione pool and its redox state in *B. fuscopurpurea*. The figure shows (**A**) reduced glutathione (GSH) content, (**B**) oxidized glutathione (GSSG) content, and (**C**) the GSH/GSSG ratio. Data are presented as means ± SD (*n* = 3). Different letters indicate significant differences among light intensities within the same treatment and time point (one-way ANOVA, Tukey’s HSD, *p* < 0.05): lowercase (a, b for 6 h; x, y for 5 d) for the −AsA group, and uppercase (A–C for 6 h; X, Y for 5 d) for the +AsA group. Asterisks (*) denote significant differences between the −AsA and +AsA treatments, while hash marks (#) indicate significant temporal differences between 6 h and 5 d under identical conditions (independent-samples *t*-test; *, # *p* < 0.05; **, ## *p* < 0.01; ***, and ### *p* < 0.001). The detailed statistical results of the three-way ANOVA regarding the main and interactive effects are provided in Table S2.

The redox state of the glutathione pool in *B. fuscopurpurea* was significantly affected by the main and interactive effects of the experimental treatments (Table S2), with exogenous AsA causing a rapid decrease in the GSH/GSSG ratio at the onset of the experiment (Figure 4). Without AsA supplementation, initial GSH levels (at 6 h) were highest under L20, while the L40 condition maintained the highest GSH level by day 5. Conversely, the highest GSSG concentration occurred under L40 at both time points. The GSH/GSSG ratio at 6 h showed a significant decrease as light intensity increased. By day 5, the ratios had declined, with the L20 group showing the lowest ratio, while the L40 group maintained the highest.

Exogenous AsA application significantly altered the glutathione pool. At 6 h, supplementation reduced GSH under L20 and L40 while increasing GSSG across all irradiances (*p* < 0.01). Consequently, the GSH/GSSG ratio fell below the respective non-supplemented controls at all light intensities. By day 5, AsA’s effects persisted, particularly on GSSG. Notably, AsA caused an increase in GSSG at L20 and L80 (*p* < 0.001). This suppressed state persisted throughout the experiment for the L20 and L40 groups, which remained at stably low ratios and were significantly lower than their non-AsA counterparts at day 5.

### 2.5. AsA-DHA Pool Dynamics and Redox State in Response to Light and AsA Treatments

**Figure 5:**
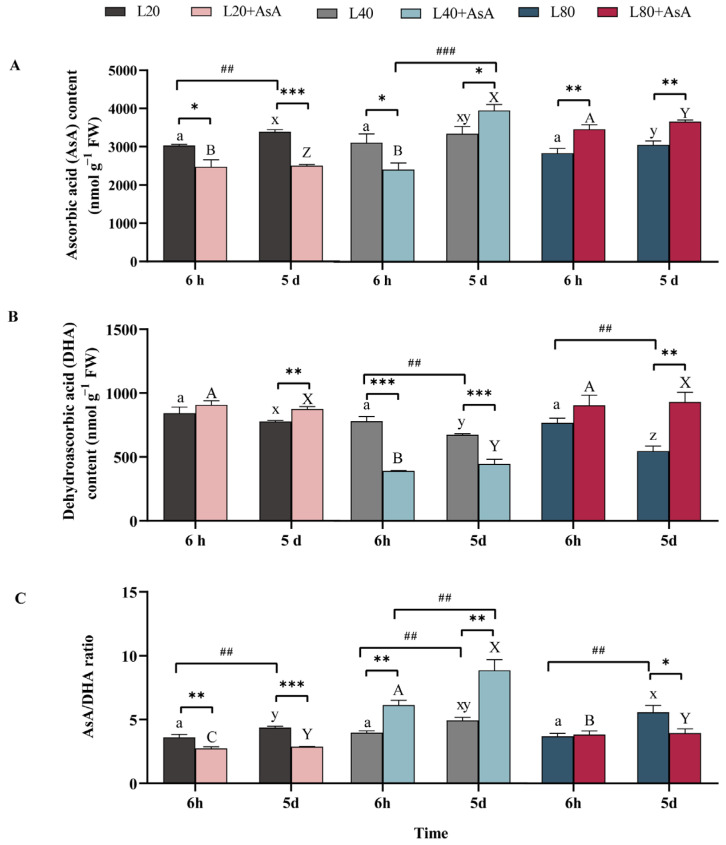
Changes in the ascorbate pool and its redox state in *B. fuscopurpurea*. The figure shows (**A**) ascorbic acid (AsA) content, (**B**) dehydroascorbic acid (DHA) content, and (**C**) the AsA/DHA ratio. Data are presented as means ± SD (*n* = 3). Different letters indicate significant differences among light intensities within the same treatment and time point (one-way ANOVA, Tukey’s HSD, *p* < 0.05): lowercase (a for 6 h; x–z for 5 d) for the −AsA group, and uppercase (A–C for 6 h; X–Z for 5 d) for the +AsA group. Asterisks (*) denote significant differences between the −AsA and +AsA treatments, while hash marks (#) indicate significant temporal differences between 6 h and 5 d under identical conditions (independent-samples *t*-test; * *p* < 0.05; **, ## *p* < 0.01; ***, and ### *p* < 0.001). The detailed statistical results of the three-way ANOVA regarding the main and interactive effects are provided in Table S2.

In contrast to the glutathione pool, the ascorbate pool exhibited gradual, long-term variations, which were highly dependent on the interactive effects of varying light intensities and AsA treatments (Table S2). Following a five-day culture, the addition of exogenous AsA resulted in the highest ascorbate redox state (AsA/DHA ratio) under the L40 condition (Figure 5). In the absence of exogenous AsA, no significant differences in ascorbate parameters were detected among the light treatments at 6 h. By day 5, however, significant light-dependent variations emerged: the DHA content was highest under L20, whereas the maximum AsA/DHA ratio was recorded under L80. The application of exogenous AsA resulted in distinct variations dependent on light intensity and exposure duration. At 6 h, AsA supplementation was associated with lower endogenous AsA levels under L20 and L40, corresponding to a decreased AsA/DHA ratio under L20. In contrast, under L80, the exogenous AsA treatment increased the endogenous AsA content (*p* < 0.01). A significant increase in the AsA/DHA ratio at this early time point was observed exclusively under L40 (*p* < 0.01). By day 5, the accumulation patterns changed. Under L20, the endogenous AsA content remained significantly lower in the supplemented group (*p* < 0.01). Conversely, under L40 and L80, AsA supplementation resulted in higher endogenous AsA levels (*p* < 0.05).

### 2.6. Activity Patterns of Key Enzymes in the AsA-GSH Cycle Under Varying Light Intensities and AsA Addition

**Figure 6:**
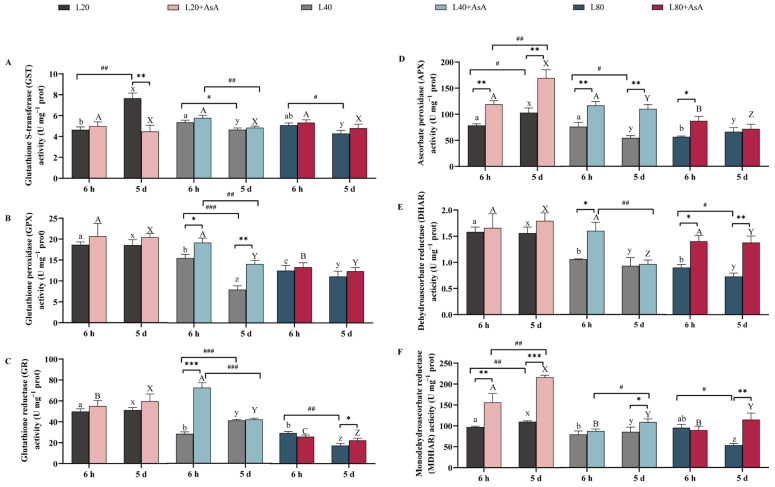
Activity of key enzymes in the AsA-GSH cycle in *B. fuscopurpurea*. The figure shows changes in the activity of (**A**) glutathione S-transferase (GST) activity, (**B**) glutathione peroxidase (GPX) activity, (**C**) glutathione reductase (GR) activity, (**D**) ascorbate peroxidase (APX) activity, (**E**) dehydroascorbate reductase (DHAR) activity, and (**F**) monodehydroascorbate reductase (MDHAR) activity. Data are presented as means ± SD (*n* = 3). Different letters indicate significant differences among light intensities within the same treatment and time point (one-way ANOVA, Tukey’s HSD, *p* < 0.05): lowercase (a–c for 6 h; x–z for 5 d) for the −AsA group, and uppercase (A–C for 6 h; X–Z for 5 d) for the +AsA group. Asterisks (*) denote significant differences between the −AsA and +AsA treatments, while hash marks (#) indicate significant temporal differences between 6 h and 5 d under identical conditions (independent-samples *t*-test; *, # *p* < 0.05; **, ## *p* < 0.01; ***, and ### *p* < 0.001). The detailed statistical results of the three-way ANOVA regarding the main and interactive effects are provided in Table S2.

The activities of the enzymes involved in the AsA-GSH cycle exhibited complex response patterns driven by the main and interactive effects of light intensity, exogenous AsA, and time (Table S2; Figure 6). While enzymes such as glutathione reductase (GR) and monodehydroascorbate reductase (MDHAR) were significantly modulated by all main and interactive effects, the interactive effect of AsA and time was not significant for GPX, APX, and dehydroascorbate reductase (DHAR). Furthermore, the overall main effect of time was not significant for glutathione S-transferase (GST) activity. In the absence of exogenous AsA, higher enzymatic activities were generally recorded under lower light conditions (such as GPX, GR, and DHAR). At 6 h, the activity of GST was highest under L40, whereas glutathione reductase (GR) and glutathione peroxidase (GPX) activities reached their maxima under L20. By day 5, the highest enzymatic activities for this group (GST, APX, and MDHAR) were observed under L20.

Exogenous AsA addition initially increased the activities of several enzymes at 6 h. Specifically, supplementation elevated GR and GPX activities under L40 and enhanced AsA-recycling enzymes, including APX across all light conditions, DHAR under L40 and L80, and MDHAR under L20. GST activity exhibited no significant changes at this initial stage. By day 5, the enzymatic response patterns had altered, characterized primarily by a significant increase in MDHAR activity across all light treatments. Additionally, higher APX and GPX activities were recorded under L40. Conversely, prolonged AsA exposure under L20 resulted in significantly lower GST activity compared to the unsupplemented condition.

### 2.7. Outlining the AsA-GSH Cycle of B. fuscopurpurea in Response to Light Intensity Stress

**Figure 7:**
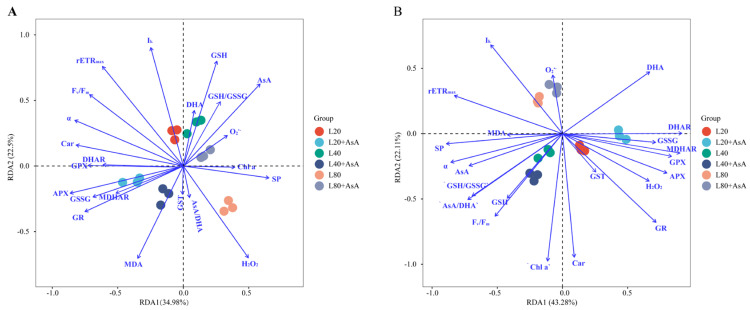
Redundancy analysis (RDA) biplots of physiological and biochemical parameters in *B. fuscopurpurea* under different sub-saturating light intensities and exogenous AsA treatments for 6 h (**A**) and 5 d (**B**). Symbols represent treatment groups, and vectors indicate the measured variables. Vector length and angles reflect the explained variance and the degree of correlation (acute: positive; obtuse: negative), respectively. AsA, ascorbic acid; DHA, dehydroascorbic acid; GSH, reduced glutathione; GSSG, oxidized glutathione; APX, ascorbate peroxidase; GR, glutathione reductase; GST, glutathione S-transferase; GPX, glutathione peroxidase; DHAR, dehydroascorbate reductase; MDHAR, monodehydroascorbate reductase; O_2_^•−^, superoxide anion; MDA, malondialdehyde; SP, soluble protein; Chl a, chlorophyll-a; Car, carotenoid; F_v_/F_m_, maximum quantum yield of photosystem II; rETR_max_, maximum relative electron transport rate; α, initial slope; and I_k_, semi-saturated light intensity.

Redundancy analysis (RDA) evaluated the relationships among parameters in *B. fuscopurpurea* under different sub-saturating light intensities and exogenous AsA treatments at 6 h and 5 d (Figure 7). At 6 h, AsA altered the physiological responses (Figure 7A). The L40 group with AsA correlated positively with MDA, APX, GR, and MDHAR. Under L20 light, the untreated control correlated with photosynthetic parameters (F_v_/F_m_ and rETR_max_). The addition of AsA removed this correlation. Instead, the L20 group with AsA correlated with GPX, DHAR, and damage indicators. Under L80 light, the untreated control correlated with H_2_O_2_ and GST. The L80 group with AsA correlated instead with antioxidant substrates (AsA, GSH, and DHA) and the ratio of GSH to GSSG. After 5 d, parameter relationships changed (Figure 7B). The redox ratios of GSH to GSSG and AsA to DHA varied together. GPX, MDHAR, and GST formed another distinct group. The L40 group with AsA maintained a positive correlation with the AsA pool, SP, and MDA. The L20 group with AsA showed no correlation with photosynthetic parameters. It correlated positively with DHA, H_2_O_2_, GSSG, and DHAR. Under L80 light, both the untreated and AsA treated groups responded similarly. The L80 group with AsA correlated with superoxide anion (O_2_^•−^) but showed no correlation with antioxidant enzymes.

**Figure 8:**
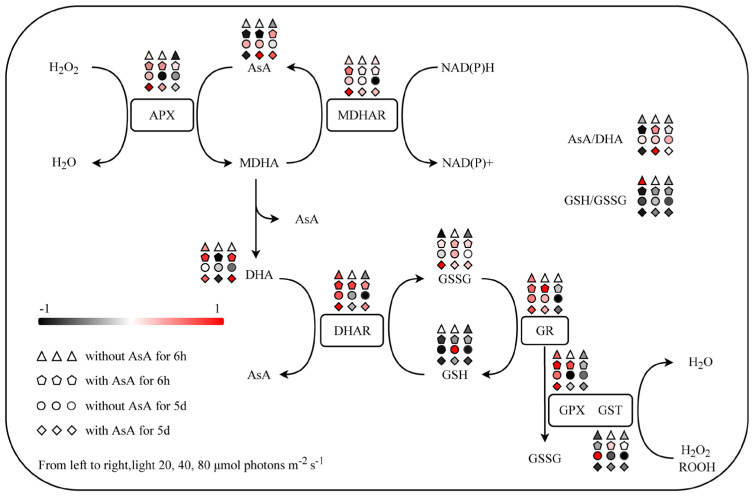
Schematic diagram of the response patterns of components in the AsA-GSH cycle in *B. fuscopurpurea*. The diagram displays the relative changes in key metabolites and enzyme activities at 6 h and 5 d under different light intensities and exogenous AsA treatments. The color code represents the normalized fold-change relative to the control group (40 µmol photons m^−2^ s^−1^, without AsA at 6 h), with red indicating an increase and black indicating a decrease.

Figure 8 integrates the response patterns of the AsA-GSH cycle using normalized fold-changes to visualize specific points of metabolic imbalance. Without exogenous AsA, the L40 condition at day 5 maintained the most stable levels of key metabolite pools (AsA, GSH) and redox ratios (AsA/DHA, GSH/GSSG).

Exogenous AsA application altered this balance. Initial supplementation at 6 h, particularly under L20 and L40, caused a marked reduction in the GSH/GSSG ratio alongside the activation of AsA-recycling enzymes such as APX and MDHAR. Long-term outcomes at day 5 diverged depending on the energy state. Under L20, the system did not restore the AsA/DHA ratio, and the GSH/GSSG ratio remained significantly reduced. Under L80, elevated MDHAR activity coincided with the restoration of the AsA/DHA ratio, while the GSH/GSSG ratio remained significantly suppressed. Conversely, the L40 condition maintained both pools at measurable levels, achieving the highest AsA/DHA ratio alongside continuous suppression of the GSH/GSSG ratio.

## 3. Discussion

### 3.1. Establishing the Physiological States Within the Sub-Saturating Light Gradient

As discussed in the Introduction, the turbid aquaculture environment exposes the bulk biomass to chronic energy limitation. To confirm that our experimental gradient accurately reflects this sub-saturating regime, rapid light curve (RLC) measurements extending up to 336 µmol photons m^−2^ s^−1^ were conducted. Specifically, rapid light curve measurements revealed that the calculated I_k_ values under the 40 µmol photons m^−2^ s^−1^ condition were approximately 190 µmol photons m^−2^ s^−1^. This physiologically confirms that our entire experimental gradient (20–80 µmol photons m^−2^ s^−1^) remained within a sub-saturating, energy-limited regime.

Within this gradient, 40 µmol photons m^−2^ s^−1^ supported the most stable physiological state for *B. fuscopurpurea* during the 5-day experiment. This was evidenced by the highest sustained levels of SP (Figure 3C) and Chl a (Figure 2A), coupled with the lowest membrane lipid peroxidation (MDA, Figure 3B) and the most stable glutathione redox state (highest GSH/GSSG ratio among controls at day 5, Figure 4C). This suggests a state where the limited energy gains from photosynthesis were optimally balanced with the metabolic requirements of maintenance [37].

Exposure to L20 exhibited characteristics of light-limited growth. The consistently lowest photosynthetic capacity (rETR_max_, Figure 1B) indicated minimal energy input, resulting in low protein accumulation. A significant decline in the GSH/GSSG ratio occurred by day 5 despite the maintenance of high pigment contents for light capture (Figure 4C), indicating an inability to sustain redox homeostasis due to insufficient reductive power [38].

At L80, the physiological stress was driven by an imbalance between energy input and utilization. Specifically, the high electron transport rate (rETR_max_) exceeded the capacity of downstream metabolic pathways to consume the produced reductants, as evidenced by the lack of increase in SP levels. This excess of excitation energy likely contributed to the observed rapid increase in H_2_O_2_ and subsequent oxidative damage by day 5. These results suggest that while L80 promotes a higher metabolic rate, the associated energy input is insufficient to offset the increased maintenance and repair requirements [37,39]. Crucially, the oxidative stress at L80 stems from a mismatch between photosynthetic electron transport and downstream metabolic capacity, rather than direct photosystem saturation. Despite operating below the I_k_, the high rETR_max_ at L80, coupled with lower SP content compared to L40, indicates a limited capacity to channel chemical energy into growth-related processes. This imbalance likely leads to the production of ROS exceeding the capacity of the antioxidant scavenging system.

### 3.2. Energy-State-Dependent Effects of Exogenous AsA Imply High Metabolic Demand

As shown in Figure 5A, the basal endogenous AsA content was approximately 3000 nmol g^−1^ FW (equivalent to an estimated intracellular concentration of 3 mM). This confirms that the 1 mM exogenous AsA applied in our treatments represents a physiologically relevant concentration.

Under the L40 condition, AsA supplementation increased the ascorbate pool and APX activity (Figure 6D), yet MDA levels continued to rise (Figure 3B). A mismatch between enzyme activation and substrate availability explains this result. This mismatch originated from an imbalance in the supply and demand of cellular NADPH. While APX was highly active, the continuous regeneration of AsA increased the requirement for NADPH [40,41]. Under restricted energy, this recycling demand consumed the limited NADPH pool and reduced its availability for GR [42,43]. This reduction caused the observed decrease in the GSH pool. Because GSH is a required substrate for GPX, its depletion restricted downstream peroxide neutralization [44]. Although absolute NADPH content and the NADPH/NADP^+^ ratio were not directly quantified in this study, the GSH/GSSG ratio serves as a robust, time-integrated physiological proxy for the cellular reductive capacity. Because glutathione regeneration is stoichiometrically coupled to NADPH oxidation via GR, the significant statistical correlation between the expansion of the AsA pool (Figure 5A), the marked decrease in the GSH/GSSG ratio (Figure 4C), and the reduction in SP (Figure 3C) provides strong indirect biochemical evidence of a redirected electron flow. This metabolic reallocation is statistically corroborated by the RDA. At 6 h, the L40 condition with AsA exhibited a strong positive correlation with MDA, APX, GR, and MDHAR (Figure 7A), indicating a rapid and pronounced activation of the antioxidant cycling system paired with early lipid peroxidation. By day 5, this group maintained a distinct positive correlation with the AsA pool and MDA (Figure 7B), further reinforcing the link between an enlarged AsA pool and sustained oxidative stress under energy limitation. Concurrently, the marked separation of the GSH pool from the AsA-supplemented groups in the RDA space highlights a critical metabolic deficit. Under sub-saturating light, the depletion of the GSH pool serves as a proxy indicator for a restricted NADPH supply, as glutathione regeneration is stoichiometrically coupled to NADPH availability. This suggests a prioritized allocation of limited reducing power toward antioxidant defense over carbon assimilation and protein synthesis. Importantly, this energy limitation also induces the pro-oxidant activity of AsA. Without adequate NADPH for regeneration, exogenous AsA cannot remain fully reduced. Consequently, oxidized intermediates such as DHA and semidehydroascorbate radicals accumulate. These intermediates participate in Fenton reactions with trace intracellular transition metals, including Fe^2+^ and Cu^2+^, to generate hydroxyl radicals. Therefore, the metabolic cost of supporting a large AsA pool under the L40 condition disrupted the redox homeostasis associated with GSH. Under this energy restriction, AsA acted as an oxidant. This oxidative activity likely contributed to the increase in MDA levels.

Under the energy limitation at L20, the marked depletion of the GSH pool suggests that the available NADPH was insufficient to support both the regeneration of exogenous AsA and basal redox homeostasis. The lack of expansion in the AsA pool (Figure 5A), concurrent with a decrease in the GSH/GSSG ratio (Figure 4C) and photosynthetic pigments (Figure 2A,B), indicates that the cellular reductive capacity did not meet the additional metabolic demand. The redundancy analysis (RDA) reflects this pattern of metabolic limitation. Over the experimental period, the AsA-supplemented group exhibited a spatial divergence from photosynthetic parameters (F_v_/F_m_, rETR_max_) within the ordination space, associating instead with oxidative stress markers (GSSG, DHA, and H_2_O_2_) (Figure 7). The absence of an O_2_^•−^ increase (Figure 3D) differentiates this state from high-light conditions, suggesting that the observed physiological changes at L20 are attributable to the depletion of reducing equivalents rather than the over-reduction of the electron transport chain.

Under L80, despite the higher incident irradiance, the physiological responses to exogenous AsA diverged significantly. By day 5, AsA supplementation at L80 resulted in the lowest measured MDA content and a significant increase in the maximum electron transport rate (rETR_max_). However, these effects were accompanied by a significant reduction in Chl a. The biochemical mechanisms of this photosynthetic pigment degradation require further investigation. Metabolic models suggest that the redox stress from exogenous AsA under energy limitation activates specific enzymatic degradation pathways. For instance, increased chlorophyllase activity directly degrades Chl a. The reduction in total carotenoids likely reflects the targeted oxidation of specific molecules. In red algae, specific carotenoids (including alpha carotene, beta carotene, lutein, and zeaxanthin) act as sacrificial antioxidants. They are rapidly consumed to neutralize reactive intermediates. Furthermore, the degradation of phycobiliproteins, the primary light harvesting antennae in red algae, likely occurs simultaneously. These combined processes restructure the photosynthetic apparatus. Therefore, future studies should quantify specific degradative enzymes, particularly chlorophyllase, and profile the pigments specific to Rhodophyta to map these degradation pathways. The transient 5.18-fold increase in O_2_^•−^ at 6 h indicates an immediate alteration in redox status following AsA addition [11,19]. The RDA reflects the response of the L80 group. At 6 h, AsA supplementation at L80 resulted in a statistical separation from H_2_O_2_ accumulation (associated with the control) and a positive correlation with antioxidant substrates (AsA, GSH, and DHA) and the GSH/GSSG ratio (Figure 7A). By day 5, the L80+AsA group showed a statistical separation from the antioxidant enzyme clusters and correlated with O_2_^•−^ (Figure 7B). We hypothesize that the reducing equivalent (e.g., NADPH) demand for ascorbate regeneration via the AsA-GSH cycle may alter electron flux. This alteration, together with the decrease in rETR_max_ observed at 6 h (Figure 1B), is consistent with a potential over-reduction of the photosynthetic electron transport chain [13].

The dynamics of O_2_^•−^ provide a clear biophysical explanation for this L40/L80 divergence, which is rooted in the cell’s underlying energy state. At L80, the high photosynthetic electron flow provided the energy base to generate an O_2_^•−^ increase when the AsA demand was induced, likely resulting in an over-reduction of the electron transport chain. Conversely, the system remained strictly energy-limited at L40 and L20 [45]. AsA addition imposed a uniform metabolic requirement, evidenced by GSH reduction, but the restricted electron transport chain lacked sufficient electron pressure to generate an O_2_^•−^ peak. Consequently, we infer that the differential response between L40 and L80 likely stems from a physiological constraint on O_2_^•−^ production under energy limitation rather than a suppressed signaling cascade. Damage at L40 proceeded via indirect metabolic impairment through GSH reduction. In contrast, a metabolic shift occurred in the L80 system. This shift was characterized by mitigated membrane damage alongside the concurrent production of O_2_^•−^ as a byproduct of high-energy metabolic stress.

Collectively, these findings distinguish our study from previous paradigms that widely view exogenous AsA solely as a stress-mitigating agent. We demonstrate that the efficacy of the AsA-GSH cycle is dependent on the cellular energy state. Under sub-saturating light, the regeneration of ascorbate imposes a substantial metabolic demand, which diverts limited reductants away from other essential pathways and alters global redox homeostasis.

### 3.3. Biochemical Adjustments in the AsA-GSH Cycle Under Energy Limitation

Dynamic adjustments in the activity of key AsA-GSH cycle enzymes occurred in response to both light intensity and AsA supplementation under overall energy limitation. Without AsA supplementation, higher enzyme activities, particularly for GR, GPX, APX, DHAR, and MDHAR, generally emerged at the lowest irradiance (L20) by day 5 (Figure 6). While higher enzyme activities were observed at L20, this does not necessarily equate to a more robust defense system, as it could also be influenced by altered enzyme turnover rates under strict energy limitation. The concurrent significant reduction of the GSH/GSSG ratio (Figure 4C) supports this, indicating a critical deficit in reductive power [45,46].

AsA supplementation induced rapid alterations in enzyme activities. APX activity increased across all light levels at 6 h (Figure 6D), indicating an immediate physiological adjustment to the increased AsA availability and associated H_2_O_2_ processing [47]. Over the five-day period, the AsA application significantly enhanced MDHAR activity across all light conditions (Figure 6F). Although MDHAR provides a more NADPH-efficient route for AsA regeneration compared to the DHAR-GR pathway [19,48], overall NADPH availability strictly constrains its functional efficacy [37]. The decrease in the GSH/GSSG ratio at 6 h after AsA addition (Figure 4C), particularly at L20 and L40, indicates the initial involvement of the DHAR-GR pathway. The NADPH requirement of this pathway appears to exceed that which is not met by the cellular GSH regeneration capacity under these sub-saturating conditions. The later increase in MDHAR activity suggests a potential shift to a regeneration route with lower GSH consumption. The RDA reflects this biochemical change. By day 5, the AsA/DHA and GSH/GSSG ratios showed a positive correlation within the ordination space. Additionally, the statistical association among MDHAR, GPX, and GST, along with their distinct statistical positioning relative to APX and GR, reflects a transition to alternative enzymatic pathways under prolonged energy limitation. The maintenance of the AsA pool corresponds to the cellular energy state.

It is necessary to address the non-significant effects in this study, as they provide insights into metabolic trade offs. For instance, AsA addition under L20 and L40 did not cause the transient O_2_^•−^ increase observed at L80 (Figure 3D). This result indicates that the electron transport chain under energy restriction lacks sufficient electron pressure to reduce oxygen, even when antioxidant sinks are altered. Additionally, certain enzymes showed no positive response. For example, GST activity showed no significant increase at 6 h. By day 5, GST activity under L20 was significantly lower in the AsA-treated group than in the control (Figure 6A). This lack of response likely reflects an energetic trade off. Under a reductant deficit, the algae may prioritize limited GSH and NADPH for primary ROS scavenging via APX and MDHAR while downregulating secondary detoxification pathways like GST. Therefore, these non-responses represent regulatory constraints driven by substrate limitation rather than physiological inertia.

Our schematic model integrates the data on redox state, enzyme activity, and oxidative damage (Figure 8). We propose that the cellular energy state controls antioxidant function. Sub-saturating light intensity determines the baseline supply of NADPH, which affects the redox ratio of GSH to GSSG. Exogenous AsA creates a metabolic demand. Under energy restriction at L20 and L40, the system lacks sufficient reductants. This activates APX and MDHAR but depletes the GSH pool. The resulting redox disruption causes oxidative damage, indicated by accumulated MDA. Conversely, under the higher energy flux at L80, the activation of AsA recycling enzymes maintains the ascorbate pool and reduces MDA. However, this condition induces alternative stress responses, including transient O_2_^•−^ generation and pigment degradation. Ultimately, this model demonstrates that antioxidant processing and oxidative damage depend on available metabolic energy.

### 3.4. Limitations and Future Directions

Although this study provides valuable physiological insights, we primarily characterized the metabolic responses of *B. fuscopurpurea* at the biochemical level. Without direct transcriptomic or proteomic evidence, the mechanisms proposed for energy limitation and metabolic balancing remain correlative rather than causal. Importantly, our model for the supply and demand of NADPH relies primarily on the dynamics of downstream metabolites, specifically the ratios of AsA to DHA and GSH to GSSG. This proposed mechanism explains the transient superoxide anion increase under the L80 and AsA treatments. However, this model currently lacks direct in vivo quantification of absolute NADPH levels and the ratio of NADPH to NADP^+^. Future investigations should integrate combined transcriptomics and targeted metabolomics to construct a comprehensive gene-protein-metabolite regulatory network. Furthermore, employing direct quantitative techniques, such as specific ELISA assays or HPLC-MS, is essential to empirically validate the precise regulatory role of the NADPH redox state. Ultimately, such efforts are essential to accurately map the signaling networks, transcriptional controls, and localized electron leakage pathways that govern reductant partitioning and ROS homeostasis [49,50].

These findings provide practical references for light management and AsA application in intensive *B. fuscopurpurea* aquaculture. We recommend an optimal light intensity of approximately 40 µmol photons m^−2^ s^−1^. This intensity balances photosynthetic efficiency with basal redox homeostasis. Raft culture systems often have high density and water turbidity. However, to prevent acute midday oxidative damage while maintaining optimal irradiance, we suggest implementing adjustable shading or dynamic cultivation depth management. Our data indicate that exogenous AsA should be used cautiously under severe energy limitation. The metabolic requirement of ascorbate regeneration can trigger oxidative effects and exacerbate lipid peroxidation. Therefore, the AsA application should be optimized. It should serve primarily as a preventive measure prior to predictable strong light stress events. This approach enhances algal antioxidant capacity and maintains commercial yields.

## 4. Materials and Methods

### 4.1. Sample Collection

The gametophytic thalli of *B. fuscopurpurea* were collected in November 2022 from a farm in Putian, Fujian, China (25°2′ N, 119°5′ E). The samples were placed in sterile, airtight plastic bags and transported to the lab in an insulated container at a temperature below 10 °C. Upon arrival, the thalli were rinsed three times with filtered seawater (0.22 µm filtration) to remove debris and surface epiphytes. Healthy thalli were selected and pre-cultured for 3 days in sterilized natural seawater enriched with 6.46 µmol L^−1^ KH_2_PO_4_-P and 142.86 µmol L^−1^ NaNO_3_-N. The pre-culture conditions were set at 40 µmol photons m^−2^ s^−1^ with a 12:12 h light–dark cycle. Following the pre-culture period, experiments with different light intensity gradients were conducted. To minimize the impact of environmental fluctuations on the physiological and biochemical measurements, culture parameters were kept stable throughout both the pre-culture and experimental periods. Specifically, cultures were maintained in an incubator at 15 ± 0.5 °C, and salinity was kept at 30 ± 1 psu. Continuous aeration was provided to keep dissolved oxygen (DO) near saturation and to stabilize the seawater pH.

### 4.2. Experimental Design

Three light intensity levels were set: 20 µmol photons m^−2^ s^−1^ (L20), 40 µmol photons m^−2^ s^−1^ (L40), and 80 µmol photons m^−2^ s^−1^ (L80). Based on our preliminary experiments, the light saturation point (I_k_) for *B. fuscopurpurea* under these culture conditions is approximately 190 µmol photons m^−2^ s^−1^. Therefore, this experimental gradient was confirmed to represent three distinct levels of sub-saturating light conditions. For each light intensity, two treatments were performed: one with 1 mmol L^−1^ AsA supplementation and the other without. The 1 mM exogenous AsA concentration was selected based on the estimated basal intracellular concentration of *B. fuscopurpurea* (~3 mM, derived from preliminary quantification of ~3000 nmol g^−1^ FW, assuming an approximate cellular water content typical for macroalgae). This concentration is below the reported stress-induced accumulation limit of 42 mM in red algae [51,52], thereby avoiding potential toxic effects. Additionally, an initial addition of 1 mM is required to offset the natural auto-oxidation of AsA in aerated seawater during the experimental period. Other conditions were identical to the culture conditions. Each treatment was performed in triplicate. Chlorophyll fluorescence parameters were measured after 6 h, 1 d, 3 d, and 5 d of incubation. Biochemical parameters were assessed after 6 h and 5 d. To minimize inter-assay variability, all commercial kits of the same type were sourced from the same manufacturing batch.

### 4.3. Measurement of Chlorophyll Fluorescence

Chlorophyll fluorescence parameters were assessed using a chlorophyll fluorescence imaging system (Maxi-Imaging-PAM, Walz, Effeltrich, Germany). After 20 min of dark adaptation, the maximum quantum yield of photosystem II (F_v_/F_m_) was measured. Rapid light curve (RLC) measurements were then conducted. Actinic light intensities of 1, 11, 21, 36, 56, 81, 111, 146, 186, 281, and 336 µmol photons m^−2^ s^−1^ were applied, with each intensity lasting for 20 s. RLC fitting analysis [53] was used to determine photosynthetic parameters, including the maximum relative electron transport rate (rETR_max_), minimum saturating light intensity (I_k_), and the initial slope of the RLC (α). The rETR_max_ represents the maximum capacity of electron transport in the photosynthetic apparatus, reflecting the overall photosynthetic efficiency under optimal light conditions. I_k_ indicates the sample’s tolerance to high light, and α measures the effectiveness of light energy use at low light intensities.

### 4.4. Pigment Estimation

Samples (0.1 g FW) were ground in 10 mL PBS (pH 6.8) and 5 mL 95% ethanol, then extracted in the dark at 4 °C for 24 h. After centrifuging (Centrifuge 5804 R; Eppendorf AG, Hamburg, Germany) at 5000× *g* for 15 min, concentrations of Chl a and Car were measured using an ultraviolet spectrophotometer (UV-180, Shimadzu, Kyoto, Japan). Chl a and Car concentrations were calculated using Wellburn’s methodology [54].

### 4.5. Measurement of H_2_O_2_, MDA, SP, and O_2_^•−^

The contents of malondialdehyde (MDA), hydrogen peroxide (H_2_O_2_), soluble protein (SP), and the production rate of superoxide anion (O_2_^•−^) were determined using commercial micro-assay kits (MDA: Cat. No. MDA-1-Y; H_2_O_2_: Cat. No. H_2_O_2_-1-Y; SP: Cat. No. BCAP-1-W; O_2_^•−^: Cat. No. SA-1-G), all purchased from Suzhou Comin Biotechnology Co., Ltd. (Room 501, Building C18, No. 218 Xinghu Street, Biobay, Suzhou, China). For all assays, fresh algal samples (0.1 g) were homogenized on ice and extracted strictly utilizing the specific extraction reagents and proprietary buffers dictated by the manufacturer’s protocols for each respective kit. For example, we used prechilled acetone for H_2_O_2_ extraction. To ensure the biochemical stability of all targets, all homogenization and initial centrifugation steps were performed strictly at 4 °C. All spectrophotometric absorbance measurements were performed using a standard 1 cm pathlength micro-cuvette. The specific procedures were as follows:

H_2_O_2_ determination: The acetone homogenate was centrifuged (8000× *g*, 10 min, 4 °C). The supernatant was reacted with titanium reagents to form a precipitate, recovered by centrifugation (4000× *g*, 10 min, 25 °C), and dissolved. Absorbance was measured at 415 nm [55].

MDA determination: The homogenate was centrifuged (8000× *g*, 10 min, 4 °C). The supernatant was mixed with the thiobarbituric acid (TBA) reagent, heated at 95 °C for 30 min, quickly cooled on ice, and centrifuged again (10,000× *g*, 10 min, 25 °C). MDA content was calculated using the absorbance difference between 532 nm and 600 nm (A532–A600) [56].

SP quantification: Following centrifugation (10,000× *g*, 10 min, 4 °C), the supernatant was mixed with the bicinchoninic acid (BCA) working solution, incubated at 60 °C for 30 min, and the absorbance was recorded at 562 nm [57].

O_2_^•−^ production rate: Based on the hydroxylamine hydrochloride method [58], the homogenate was centrifuged (10,000× *g*, 20 min, 4 °C). The supernatant was incubated with specific reagents at 37 °C for 20 min, followed by centrifugation (8000× *g*, 5 min, 25 °C). The absorbance of the resulting red azo compound was measured at 530 nm.

### 4.6. Measurement of the Metabolites and Antioxidant Enzymes in the AsA-GSH Cycle

All biochemical assays were performed using reagent kits (AsA: Cat. No. ASA-2-W; DHA: Cat. No. DHA-1-G; GSH: Cat. No. GSH-1-W; GSSG: Cat. No. GSSG-1-W; GR: Cat. No. GR-1-W; GPX: Cat. No. GPX-1-Y; APX: Cat. No. APX-1-W; DHAR: Cat. No. DHAR-1-W; MDHAR: Cat. No. MDHAR-1-W; GST: Cat. No. GST-1-W; Suzhou Comin Biotechnology, Suzhou, China) according to the manufacturer’s instructions. AsA and DHA contents were measured using chromogenic reactions and absorbance at 420 nm for AsA [59] and at 265 nm for DHA [60]. DHAR activity was assessed by measuring the reduction of DHA, with absorbance changes measured at 265 nm. MDHAR activity was determined by monitoring the rate of NADPH oxidation, reflected by a decrease in absorbance at 340 nm.

Briefly, total GSH (T-GSH, representing the sum of reduced GSH and GSSG) was quantified spectrophotometrically at 412 nm. The assay mixture contained 100 µL of the extracted sample, 300 µL of yeast-derived glutathione reductase (GR, 5 U), 300 µL of 5,5′-dithiobis-(2-nitrobenzoic acid) (DTNB, 3 mM), and 300 µL of NADPH (0.5 mM). The content of GSSG was determined using the same method following pre-treatment of the extract with 2-vinylpyridine at 27 °C for 1 h to derivatize GSH, thereby selectively quantifying GSSG. The GSH concentration was calculated by subtracting the measured GSSG from the total GSH content. A standard curve prepared with known concentrations of GSH was used for quantification. GR activity was measured by the rate of NADPH dehydrogenation, indicated by the absorbance at 340 nm.

GST activity was determined using the method of Habig [61], by monitoring the increase in absorbance at 340 nm due to the conjugation of GSH with 1-chloro-2,4-dinitrobenzene (CDNB). GPX activity was evaluated by measuring the consumption of GSH in the enzymatic reaction, with absorbance recorded at 412 nm [62]. APX activity was determined based on absorbance changes at 290 nm, reflecting the reduction of hydrogen peroxide [47].

### 4.7. Data Analysis

All data are presented as the mean ± standard deviation (SD) of three biological replicates (*n* = 3). Statistical analyses were performed using SPSS software(version 25; IBM Corp., Armonk, NY, USA). Prior to analysis, variance homogeneity was confirmed using the median-based Levene’s test. A three-way analysis of variance (ANOVA) was initially used to assess the main and interactive effects of light intensity, exogenous AsA supplementation, and exposure time on the measured parameters (Tables S1 and S2). When significant interactive effects were identified, the data were partitioned to evaluate simple main effects. Specifically, a one-way ANOVA followed by Tukey’s HSD post hoc test was used to compare differences among the three light intensities within matched AsA treatments and time points. Furthermore, independent-samples t-tests were used to determine the specific effects of AsA addition (+AsA vs. −AsA) and to compare temporal differences under identical experimental conditions. The threshold for statistical significance was set at *p* < 0.05. All graphical representations were generated using GraphPad Prism(version 8.0.2; GraphPad Software, San Diego, CA, USA). Redundancy analysis (RDA) was performed using R software (version 4.2.0; R Core Team, Vienna, Austria) with the ‘vegan’ package.

## 5. Conclusions

This study evaluated the physiological responses of *B. fuscopurpurea* to sub-saturating irradiances and exogenous AsA supplementation. Under unsupplemented conditions, a moderate sub-saturating light level (40 µmol photons m^−2^ s^−1^) provided an optimal balance, sustaining internal redox homeostasis and maximizing protein synthesis. Conversely, energy limitation at 20 µmol photons m^−2^ s^−1^ constrained both basic physiological maintenance and biomass accumulation. Although the 80 µmol photons m^−2^ s^−1^ treatment remained below the saturation point (I_k_), it coincided with a transient accumulation of H_2_O_2_ and subsequent elevated MDA compared to the 40 µmol photons m^−2^ s^−1^ condition, indicating a mismatch between photosynthetic electron transport and downstream utilization capacity. The addition of AsA yielded differential effects depending on the light environment. Under strict energy limitation at 20 µmol photons m^−2^ s^−1^, supplementation exacerbated redox imbalance. At 80 µmol photons m^−2^ s^−1^, AsA reduced lipid peroxidation and maintained electron transport capacity, but these adjustments occurred alongside significant reductions in soluble protein and pigment content. This pattern suggests that under conditions of metabolic limitation, the NADPH requirement for AsA regeneration may limit the resources available for biosynthetic pathways. Therefore, in turbid aquaculture environments, the efficacy of antioxidant supplementation appears constrained by the algal energy status.

## Data Availability

Data are contained within the article.

## Supplementary Materials

The following supporting information can be downloaded at https://www.mdpi.com/article/10.3390/plants15081165/s1, Table S1. Three-way ANOVA (*F*-values) of the main and interactive effects of light intensity (L), AsA treatment (A), and time (T) on chlorophyll fluorescence parameters in *Bangia fuscopurpurea*. Table S2. Three-way ANOVA (*F*-values) of the main and interactive effects of light intensity (L), AsA treatment (A), and time (T) on biochemical parameters and enzyme activities in *B. fuscopurpurea*.
