# Supplementary material for: The Metabolic Regulation of Antioxidant Defense: Exogenous Ascorbate Disrupts Redox Homeostasis Under Energy Limitation in Bangia fuscopurpurea
Source: Plants (Basel). 2026 Apr 9;15(8):1165. doi: 10.3390/plants15081165 (PMC13120471; doi:10.3390/plants15081165)
Supplement: Supplementary file 1 [file plants-15-01165-s001.zip › plants-4213944-supplementary.pdf]

Table S1. Three-way ANOVA (*F*-values) of the main and interactive effects of light intensity (L), AsA treatment (A), and time (T) on chlorophyll fluorescence parameters in *Bangia fuscopurpurea*.

Source of Variation	*df*	F_v/F_m	$rETR_{max}$	I_k	α
iIiMain effects					
Light (L)	2	614.49***	300.92***	17.54***	601.85***
AsA (A)	1	557.25***	315.07***	0.06	817.40***
Time (T)	3	207.56***	65.112***	82.92***	87.00***
Interactions					
L × A	2	141.76***	252.17***	51.87***	177.73***
L × T	6	51.04***	141.82***	34.77***	86.89***
A × T	3	46.59***	87.75***	92.35***	117.97***
L × A × T	6	22.70***	33.81***	15.96***	54.49***

Note: *df*, degrees of freedom. F_v/F_m, maximum quantum yield of photosystem II; $rETR_{max}$, maximum electron transport rate; I_k, semi-saturated light intensity; α, initial slope. Asterisks denote statistical significance: * $p < 0.05$, ** $p < 0.01$, *** $p < 0.001$.

Table S2. Three-way ANOVA (*F*-values) of the main and interactive effects of light intensity (L), AsA treatment (A), and time (T) on biochemical parameters and enzyme activities in *B. fuscopurpurea*.

Source of variation	Light (L)	AsA (A)	Time (T)	L × A	L × T	A × T	L × A × T
df	2	1	1	2	2	1	2
**pigments**							
Chl a	129.16***	111.37***	2258.06***	63.29***	462.24***	136.75***	124.30***
Car	458.32***	13.17**	1231.33***	14.80***	166.40***	41.63***	76.75***
**Oxidative stress & Soluble protein**							
H_2O_2	5.3*	8.61**	0.025	23.01***	91.52***	9.36**	48.35***
MDA	31.29***	13.17**	168.20***	819.14***	105.90***	75.17***	230.27***
SP	184.06***	42.82***	10.48**	4.92*	16.38***	2.34	0.31
$O_2^{\cdot -}$	1616.47***	1579.94***	1686.03***	2028.17***	1358.59***	1092.64***	1669.44***
**AsA-GSH cycle metabolites**							
GSH	24.75***	6.76*	15.36**	10.71***	7.21**	3.90	5.87**
GSSG	201.35***	1047.51***	137.58***	563.59*	4.76*	25.18***	4.40*
GSH/GSSG	15.59***	60.99***	55.42***	22.87***	7.38**	24.70***	10.32**
AsA	30.86***	1.28	90.32***	72.33***	25.81***	12.68**	30.85***
DHA	143.97***	0.64	16.82***	145.41***	2.29	27.94***	4.87*
AsA/DHA	163.67***	9.47**	85.41***	127.46***	11.70***	0.83	19.26***
GST	8.59**	4.60*	0.42	28.35***	36.00***	26.99***	28.62***
GPX	98.87***	36.81***	34.38***	7.10**	18.93***	1.08	0.82
GR	279.34***	85.56***	17.54***	36.26***	12.13***	24.27***	58.15***
APX	99.14***	210.54***	6.22*	16.29***	32.40***	0.92	7.96**

DHAR	63.65***	59.52***	10.19**	7.90***	8.39***	0.61	6.31**
MDHAR	126.80***	163.64***	18.34***	39.17***	15.39***	43.29***	5.42*

Note: *df*, degrees of freedom; Chl a, chlorophyll-a; Car, carotenoid; H_2O_2, hydrogen peroxide; MDA, malondialdehyde; SP, soluble protein; $O_2^{\bullet-}$, superoxide anion; GSH, reduced glutathione; GSSG, oxidized glutathione; AsA, ascorbic acid; DHA, dehydroascorbic acid; GST, glutathione S-transferase; GPX, glutathione peroxidase; GR, glutathione reductase; APX, ascorbate peroxidase; DHAR, dehydroascorbate reductase; MDHAR, monodehydroascorbate reductase. Asterisks denote statistical significance: * $p < 0.05$, ** $p < 0.01$, *** $p < 0.001$.
